# Sustained Microglial Activation Promotes Synaptic Loss and Neuronal Dysfunction after Recovery from ZIKV Infection

**DOI:** 10.3390/ijms25179451

**Published:** 2024-08-30

**Authors:** Nahyun Kim, Hanul Choi, Uijin Kim, Suyeon Kim, Young Bong Kim, Ha Youn Shin

**Affiliations:** 1 Department of Biomedical Science & Engineering, Konkuk University, Seoul 05029, Republic of Korea; knh64@naver.com (N.K.); rladmlwls135@naver.com (U.K.); 0924tndus123@naver.com (S.K.); 2 Department of Bio-Industrial Technologies, Konkuk University, Seoul 05029, Republic of Korea; chlgksmf9977@hanmail.net

**Keywords:** ZIKV, long-term effects, microglial activation, neuroinflammation, synaptic loss

## Abstract

Zika virus (ZIKV), transmitted by Aedes mosquitoes, has been a global health concern since 2007. It primarily causes fetal microcephaly and neuronal defects through maternal transmission and induces neurological complications in adults. Recent studies report elevated proinflammatory cytokines and persistent neurological alterations post recovery, but the in vivo mechanisms remain unclear. In our study, viral RNA loads in the brains of mice infected with ZIKV peaked at 7 days post infection and returned to baseline by day 21, indicating recovery. RNA sequencing of the cerebral cortex at 7 and 21 days revealed upregulated genes related to neuroinflammation and microglial activation. Histological analyses indicated neuronal cell death and altered neurite morphology owing to severe neuroinflammation. Additionally, sustained microglial activation was associated with increased phospho-Tau levels, constituting a marker of neurodegeneration. These findings highlight how persistent microglial activation leads to neuronal dysfunction post ZIKV recovery, providing insights into the molecular pathogenesis of ZIKV-induced brain abnormalities.

## 1. Introduction

Zika virus (ZIKV), a member of the Flaviviridae family within the genus Flavivirus, was first identified in the Zika forest of Uganda in 1947 [1]. Since the discovery of the first human infection in 1950, ZIKV has spread from Africa to Asia and subsequently to the Americas, causing significant epidemics until 2016, with sporadic cases still reported worldwide. Primarily transmitted through the bites of Aedes mosquitoes, ZIKV can also spread through sexual contact, blood transfusion, or mother-to-child transmission during pregnancy. Although ZIKV infection typically results in mild symptoms, such as fever, rash, headache, joint pain, and red eyes lasting several days to a week, it poses significant risks during pregnancy. Infection during pregnancy can lead to severe birth defects including microcephaly and neurological conditions like myelitis and encephalitis [2,3]. These neurological complications are not limited to congenital cases but also affect adults following ZIKV infection [4,5]. Studies have demonstrated elevated levels of proinflammatory cytokines, including interleukin (IL)-1β, interferon (IFN)-γ, IL-6, and IL-8, in ZIKV-infected adult patients compared with healthy individuals [6]. Additionally, ZIKV infection has been linked to the induction of Guillain–Barre syndrome, an autoimmune neurological disease [7]. Furthermore, ZIKV has been detected in adult human brain tissue [8]. ZIKV infection has also been increasingly associated with CNS pathology in adults, including acute myelitis, encephalitis, meningoencephalitis, and encephalomyelitis [4,5,9,10]. Notably, a clinical study found that ~40% of ZIKV-infected patients still exhibited neurological changes 1 year after symptom onset, with some patients showing incomplete recovery even after 2 years [11]. Collectively, these findings underscore the detrimental effects of ZIKV infection on the mature nervous system and suggest potential long-term neurological consequences, yet the precise underlying mechanisms remain to be fully elucidated.

One of the primary cell types targeted by ZIKV within the brain comprises glial cells [12,13,14]. Among glial cells, microglia—the predominant innate immune cells in the central nervous system (CNS)—play a crucial role in neurodevelopment and CNS homeostasis [15]. Responsible for maintaining normal brain function and tissue integrity, activated microglia serve as the initial defense against invading pathogens, and their activation leads to the increased production of proinflammatory cytokines, chemokines, and reactive oxygen species (ROS) [16,17]. Although microglial activation is typically considered protective for the brain, prolonged activation can cause chronic inflammation, leading to irreversible CNS damage. Such persistent neuroinflammation not only impairs neuronal plasticity and memory but also contributes significantly to tissue damage in various neurodegenerative disorders [16,18]. In this context, microglial activation has been linked to the increased phosphorylation of the Tau protein, exacerbating Tau pathology—a hallmark of neurodegenerative diseases such as Alzheimer’s disease [19]. In addition to the pathological accumulation of neurofibrillary tangles (NFTs) containing the hyperphosphorylated Tau protein, Alzheimer’s disease is characterized by the accumulation of amyloid-β (Aβ) plaques and neuronal loss [20,21]. The accumulation of Aβ triggers microglial responses, promoting Tau protein hyperphosphorylation and NFT formation, ultimately leading to neurodegeneration and cognitive decline [22].

While several studies have reported neuroinflammation and microglial activation as consequences of ZIKV infection [23,24], the detailed mechanisms underlying brain impairments in recovering patients remain elusive. This may be attributable to the lack of a suitable animal model representing the recovery state post ZIKV infection. Evidence suggests that ZIKV can persist latently in mature neural cells [25], and its association with widespread changes in gene expression in the brain has been demonstrated [4,5,6]. Thus, it is crucial to move beyond acute phase symptoms to develop an understanding of the long-term neurological sequelae of ZIKV infection, including their potential to lead to neurodegenerative disorders. To this end, we established an in vivo recovery model of ZIKV infection using ZIKV-susceptible A129 mice. Through comprehensive RNA-sequencing (RNA-seq)-based transcriptome analysis, we explored gene expression profiles in the brains of recovered mice post ZIKV infection. Gene Ontology (GO) analysis revealed the significant upregulation of the biological process categories “neuroinflammatory response” and “microglial cell activation” in ZIKV-infected mice. Our findings indicate severe inflammatory pathology, neuroinflammation-induced neuronal cell death, and alterations in neurite morphology in mice recovered from ZIKV infection. Furthermore, increased levels of phospho-Tau (p-Tau) were observed together with microglial activation in recovered mouse brains. These findings shed light on the mechanism by which ZIKV affects the brain and underscore the role of this virus-induced microglial activation mechanism in neurodegeneration. They also have implications for understanding the molecular pathogenesis of ZIKV-induced abnormalities in the brain and may guide the development of effective therapeutic strategies against ZIKV infection.

## 2. Results

### 2.1. Immunodeficient A129 Mice Recover from Infection with the ZIKV-FLR Strain after 21 Days

**Figure 1:**
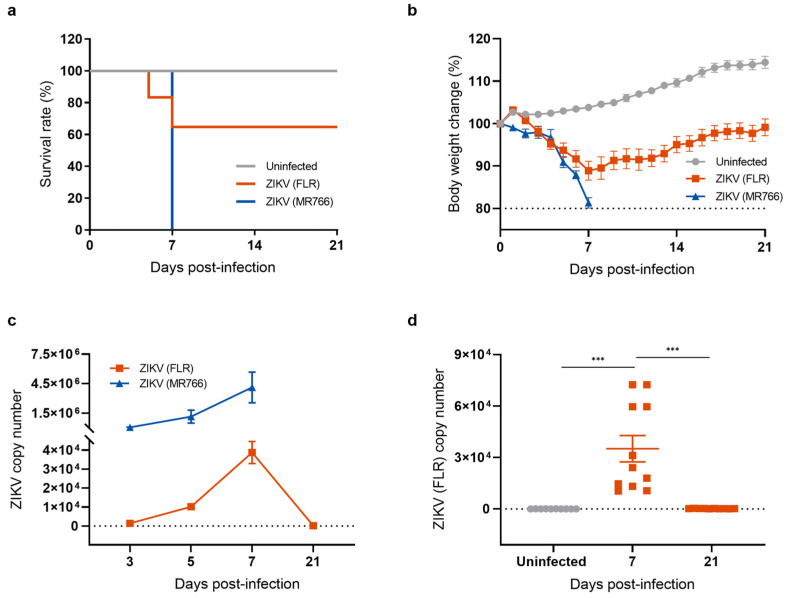
Generation of a mouse model of recovery from ZIKV infection. (**a**) Survival rates of mice used as uninfected controls and mice infected with ZIKV strains FLR (American and Asian strain) or MR766 (African strain). Survival of mice was monitored for 21 days. All mice infected with the ZIKV-MR766 strain succumbed within 7 dpi whereas fewer than 40% of mice infected with the ZIKV-FLR strain died, with over 60% surviving beyond 21 days. (**b**) Body weight change (percent) after ZIKV infection. (**c**) Comparison of ZIKV copy number, measured by RT-qPCR. (**d**) Quantification of ZIKV (FLR) RNA levels in the mouse brain using RT-qPCR analysis. All data represent means ± SEMs from at least 7 mice (*** *p* < 0.001). The results for the uninfected control are shown in gray, the ZIKV MR766 strain in blue, and the ZIKV FLR strain in red. The dashed line indicates the basal level.

To investigate the long-term effects of neuroinvasive ZIKV infection, we established a mouse model for studying the recovery phase following ZIKV infection. Type I interferon (α/β)-receptor-deficient A129 mice, which are highly susceptible to ZIKV infection, were intraperitoneally injected with 1 × 10^4^ PFU of either the ZIKV-FLR (American and Asian lineage) or the ZIKV-MR766 strain (African lineage). Mice were monitored daily for signs of illness including changes in body weight and mortality. All mice infected with the ZIKV-MR766 strain lost body weight, quickly succumbed to infection, and reached clinical endpoints within 7 days (Figure 1a). The pattern following ZIKV-FLR infection was different. Approximately 40% of infected mice succumbed to infection within 7 days and exhibited neurological signs such as hind limb paralysis whereas the remaining 60% of ZIKV-FLR-infected mice gradually regained body weight and recovered from hind limb paralysis within 21 days (Figure 1b). To examine the impacts of ZIKV-MR766 and ZIKV-FLR strains on the mouse brain, we obtained cerebral cortices from mice in each group and measured viral load by quantitative reverse-transcription polymerase chain reaction (RT-qPCR). Mice infected with ZIKV-MR766 exhibited approximately 100-fold higher levels of ZIKV RNA in the brain relative to mice infected with ZIKV-FLR, and viral replication remained active until death (Figure 1c). In contrast, viral RNA levels in ZIKV-FLR-infected mice gradually increased, peaking at 7 days post infection (dpi), and then declined to undetectable levels, indicating that the mice were free of viral replication by 21 dpi (Figure 1d). To further investigate the presence of any remaining viral proteins in cells, we performed a Western blot analysis of the ZIKV envelope (E) protein, a critical structural component of the virus that plays a central role in viral entry into host cells. The expression of the ZIKV E protein indicates an active viral presence, with peak levels typically occurring during the acute phase of infection [26,27]. Our results showed that ZIKV E protein levels increased at 7 dpi, declined to undetectable levels by 21 dpi, and remained undetectable at 28 dpi (Figure S1). On the basis of these results, we selected the relatively mild ZIKV-FLR strain to establish a mouse model of a post-ZIKV-infection recovery state.

### 2.2. The Transcriptional Profile of the Mouse Brain Is Altered after Recovery from ZIKV Infection

**Figure 2:**
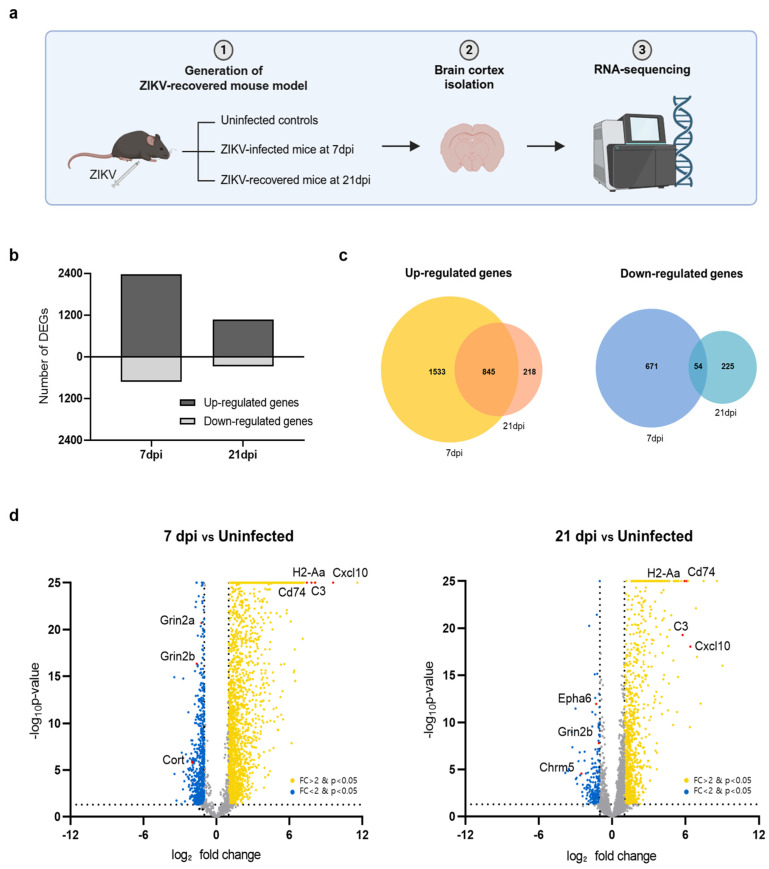
Transcriptome profiles of the cerebral cortex in A129 mice after ZIKV infection. (**a**) Schematic showing the experimental strategy for analyzing changes in gene expression in the mouse brain following ZIKV infection. (**b**) Bar chart displaying the total number of upregulated and downregulated DEGs in mouse groups harvested at 7 dpi and 21 dpi (uninfected, n = 3; 7 dpi, n = 3; 21 dpi, n = 3). Gene expression levels in both groups were normalized to those in uninfected controls. (**c**) Venn diagrams showing the overlap of upregulated genes (left) and downregulated genes (right) in mice brains harvested at 7 dpi or 21 dpi, identifying genes commonly regulated at both time points. (**d**) Volcano plots depicting gene transcripts in ZIKV-infected mouse brains harvested at 7 dpi and 21 dpi compared with uninfected controls. Yellow dots: genes upregulated upon ZIKV infection; blue dots: genes downregulated upon ZIKV infection. Selected DEGs have been highlighted in red. Gray dots indicate genes with no significant change in expression. The dashed lines represent the significance thresholds for both *p*-value and log2-fold change.

Using the ZIKV-FLR strain, we first examined whether transcriptional changes occurred in the brain tissue in ZIKV-infected mice and whether these changes persisted after recovery. The brain, particularly the cerebral cortex, is well known as a major target organ of ZIKV [8,28]. To compare transcriptome profiles between acute infection and the recovery stage, we performed RNA-seq on the cerebral cortices of ZIKV-FLR-infected mice, obtained at 7 and 21 days post infection (dpi) (Figure 2a). The cerebral cortices of A129 mice obtained at 7 dpi, exhibiting the highest viral load, were used to investigate the transcriptional profile of acute infection whereas the cerebral cortices at 21 dpi, exhibiting viral clearance, were used to examine changes in gene expression during the recovery phase. Differentially expressed genes (DEGs) in the cerebral cortex at 7 dpi and 21 dpi in ZIKV-infected mice were identified based on their normalized expression relative to that in uninfected controls. At 7 dpi, 2378 genes were upregulated and 725 genes were downregulated compared with uninfected controls; in contrast, 1063 genes were upregulated and 279 genes were downregulated at 21 dpi (Figure 2b).

We then investigated genes that were significantly up- or downregulated in common in both acute (7 dpi) and recovery (21 dpi) phases. Utilizing a Venn diagram analysis, we found that the predominant pattern was upregulation, with upregulated 845 genes and only 54 downregulated genes at both time points (Figure 2c). Among the top 20 significantly upregulated genes in both conditions were *Cxcl10*, *H2-Aa*, *C3,* and *CD74*, as illustrated in volcano plots (Figure 2d). These genes, which are known to be associated with neuroinflammation, play critical roles in regulating the immune system and inflammatory responses. Conversely, the most highly downregulated genes in common were *Grin2b, Cort*, and *Chrm5*, which function in neurotransmission. Overall, of the 3103 genes whose transcriptional expression levels changed upon ZIKV infection, more than half recovered to levels comparable to those in uninfected condition by 21 dpi whereas the remainder remained up- or downregulated even after recovery. Notably, genes related to neuroinflammation were activated whereas those related to neurotransmission were repressed.

### 2.3. ZIKV-Induced Microglial Cell Activation Results in Upregulation of Neuroinflammatory Responses and Downregulation of Neurotransmission

**Figure 3:**
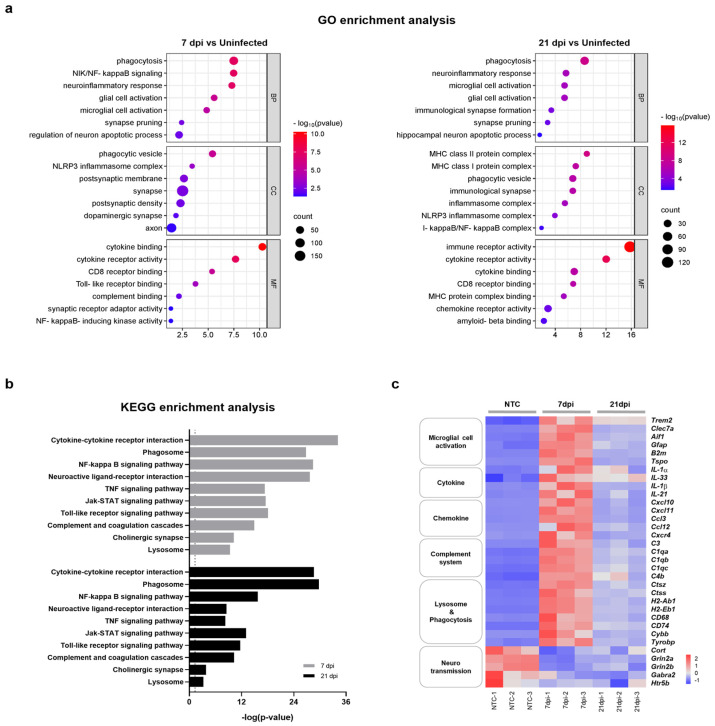
Functional categorization of DEGs in the brains of mice infected with and recovered from ZIKV. (**a**) Selected GO functional classifications for significant DEGs in ZIKV-infected mice brains obtained at 7 and 21 dpi. Biological processes associated with significant DEGs mainly included ‘microglial cell activation’, ‘neuroinflammatory responses’, ‘synapse pruning’, and ‘neuronal apoptotic process’. GO: Gene Ontology; BP: biological process; CC: cellular component; MF: molecular function. (**b**) Selected KEGG pathways associated with significant DEGs in ZIKV-infected mice brains obtained at 7 and 21 dpi. Significant pathways of common DEGs included ‘cytokine-cytokine receptor interaction’, ‘phagosome’, ‘complement and coagulation cascade’, and ‘neuroactive ligand-receptor interaction’. The vertical line represents the threshold for a *p*-value of 0.05. (**c**) Heatmap displaying the relative expression levels of significantly changed genes related to neuroinflammation (microglial cell activation, cytokine, chemokine, complement system, and lysosome and phagocytosis) and neurotransmission.

To better understand the relationship between functional pathways and DEG datasets obtained at 7 and 21 dpi, we conducted an unbiased enrichment analysis using Gene Ontology (GO) and the Kyoto Encyclopedia of Genes and Genomes (KEGG). The GO analysis of biological process (BP) terms revealed consistent enrichment of genes related to neuroinflammatory responses and microglial cell activation in brain tissues obtained at both 7 dpi and 21 dpi (Figure 3a). Significantly correlated BP terms included ‘phagocytosis’, ‘neuroinflammatory response’, ‘microglia cell activation’, ‘glial cell proliferation’, ‘synapse pruning’, and ‘regulation of neuron apoptotic process’. DEG datasets correlated with molecular function (MF) and cellular component (CC) terms also indicated a close relation to neuroinflammatory processes and the activation of microglia. DEG datasets obtained at 7 and 21 dpi were also significantly correlated with specific KEGG pathways subsets, including ‘cytokine-cytokine receptor interaction’, ‘phagosome’, ‘complement and coagulation cascades’, ‘neuroactive ligand-receptor interaction’, ‘NF-κB signaling pathway’, and ‘synapse’ (Figure 3b). Accordingly, the majority of enriched terms found in both GO and KEGG analyses were related to neuroinflammatory response, microglia cell activation, and neurotransmission. Consistent with previous reports, these results demonstrate that ZIKV infection has the potential to activate microglia and induce neurological damage [23,24]. Importantly, these results also suggest that microglial activation and neuronal damage persist after recovery from ZIKV infection. 

Microglia are resident macrophages that account for approximately 10–15% of cells in the mouse brain and function as a first-line defense system in the CNS [29]. Several studies have shown that microglial cells are permissive for ZIKV infection and that microglial cell activation results in neuroinflammation and is detrimental to neurotransmission [24,30,31,32]. To further clarify whether ZIKV-induced aberrant microglial activation correlates with neurological complications, we investigated DEGs associated with microglial activation, neuroinflammation, and neurotransmission at both 7 dpi and 21 dpi. A heatmap analysis revealed that genes associated with microglial activation and neuroinflammation, including cytokine, chemokine, complement-system, and phagocytosis genes, were significantly upregulated at 7 dpi, exhibiting up to a 10-log2-fold change (Figure 3c and Figure S2). The expression of these genes was reduced ~2-fold at 21 dpi but did not fully return to the levels of uninfected controls. On the other hand, genes associated with neurotransmission were significantly downregulated at 7 dpi and remained at similarly reduced levels at 21 dpi. These results suggest that abnormal microglial activation can persist even after recovery from ZIKV infection. This prolonged activation may contribute to ongoing neuroinflammation and potentially lead to lasting damage to neurotransmission, even in the absence of a detectable viral presence.

### 2.4. ZIKV-Induced Microglial Cell Activation Enhances Synaptic Pruning through the Complement System

Next, we sought to determine how ZIKV infection alters gene expression patterns and induces neurotoxic inflammation through microglial activation and potentially leads to neurodegeneration. Transcriptome profiling in ZIKV-infected and recovered mice revealed the sustained upregulation of the microglia cell activation markers, Clec7a, Aif1, Gfap, and Trem2 (Figure S2). As shown in Supplementary Figure S2, ZIKV significantly increased the expression of complement-related genes (C3, C1q, C4b, and C3ar1), together with genes related to microglial-mediated phagocytosis (Ctss, Ctsc, Cybb, CD74,CD68, H2-Ab1, and H2-Aa), in the brains of ZIKV-infected and recovered mice. Recent studies suggest that microglia-mediated synapse removal can be triggered by aging or disease conditions. In the CNS, microglia rely on classical complement cascades to mediate phagocytic signaling for the removal of excess synapses [18]. In Alzheimer’s mouse models, activated microglia engage in synaptic phagocytosis, a process dependent on complement factors like C3, C1q, and CR [33]. This microglial pruning of synaptic terminals is a key mechanism underlying synapse loss in neurodegenerative disorders and viral infections. To explore this further, we investigated whether mice recovering from ZIKV infection exhibited impaired synaptic function. As seen in Figure 3a, ‘synaptic pruning’ was among the most significantly expressed categories in GO analyses following ZIKV infection in mice. We found that ZIKV significantly downregulated the expression of genes encoding neuropeptides or neurotransmission receptors, such as Cort, Gabra2, Grin2b, and Htr5, in the mouse brain cortex. Genes encoding nicotinic acetylcholine receptor subunits (Chrnb4 and Chrna7), adrenoceptors (Adra2b and Adra1d), muscarinic acetylcholine receptors (Chrm1 and Chrm3), and two serotonin receptors (Htr2a and Htr4), which typically participate in the regulation of cognitive function, were downregulated at both 7 dpi and 21 dpi compared with uninfected mice brains (Figure S2). Most of these genes have been shown to modulate synaptic plasticity [34,35,36,37,38] and CNS development [39,40].

**Figure 4:**
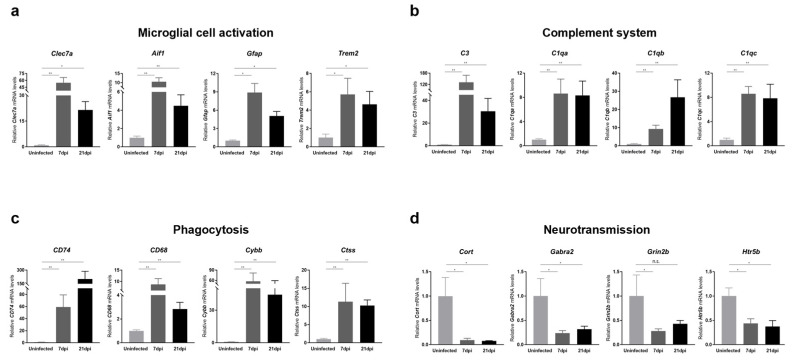
Transcriptional analysis of genes related to microglial activation, complement system, phagocytosis, and neurotransmission in the brains of ZIKV-infected and recovered mice. RT-qPCR analyses of genes associated with (**a**) microglial cell activation, (**b**) the complement system, (**c**) phagocytosis, and (**d**) neurotransmission are shown. mRNA levels of target genes were normalized to *Gapdh* mRNA levels. All data represent the relative expressions of target genes normalized to those of uninfected controls. Results are shown as means ± SEMs of six independent replicates (* *p* < 0.05; ** *p* < 0.01; n.s.: not significant).

To further validate RNA-seq data, we also performed RT-qPCR analyses on mouse cerebral cortices obtained at 7 and 21 dpi for representative genes involved in microglial cell activation, cytokine and chemokine responses, complement system activation, phagocytosis, and neurotransmission. We found that the expression of a panel of genes known to be specifically induced by microglial activation, including Clec7a, Aif1, Gfap, and Trem2, was significantly increased upon ZIKV infection (Figure 4a). Additionally, genes encoding cytokines and chemokines, including IL-1α, Il-1β, Cxcr4, and Cxcl10, were significantly upregulated in response to ZIKV infection, suggesting the presence of neuroinflammation (Figure S3). Together with microglial-activation-associated genes, genes related to complement activation, lysosomal function, and phagocytosis were significantly upregulated (Figure 4b,c). Importantly, all of these genes exhibited persistent upregulation at 21 dpi (i.e., recovery phase), and these changes were statistically significant. Notably, the C1q and C3 genes showed more than an 8-fold change in expression in ZIKV-recovered mice compared with uninfected controls. These results were consistent with the RNA-seq data (Figure S2) and suggest that ZIKV-induced microglial activation contributes to the transcriptional upregulation of genes related to complement activation, lysosomal function, and phagocytosis in the brains of ZIKV-infected and recovered mice. We also confirmed the downregulation in common in the neurotransmission-related genes, Cort, Grin2b, Gabra2, and Htr5 (Figure 4d), validating a potential contribution of microglial phagocytosis to synaptic pruning. 

Furthermore, we investigated the long-term effects of ZIKV infection on the mouse brain. At 28 dpi, the ZIKV viral protein (E) was undetectable in the brain (Figure S1), indicating viral clearance. However, RT-qPCR analyses of cerebral cortices collected at 28 dpi revealed that microglial activation remained elevated (Figure S4a). Along with persistent microglial activation, we observed the significant upregulation of genes associated with the complement system, lysosomal function, and phagocytosis (Figure S4b,c). Importantly, these genes continued to show upregulation at 28 dpi. Conversely, neurotransmission-related genes were notably downregulated (Figure S4d).

Taken together, our data suggest that the aberrant activation of microglia and complement pathways persists in both ZIKV-infected and ZIKV-recovered mice, ultimately leading to synaptic loss. These findings support our hypothesis that ZIKV-induced microglial cell activation contributes to increased synapse pruning and may explain the sustained synaptic impairment observed following long-term recovery from ZIKV infection.

### 2.5. Sustained Microglial Cell Activation after Recovery from ZIKV Infection Induces Neurotoxicity and Neuronal Cell Death

**Figure 5:**
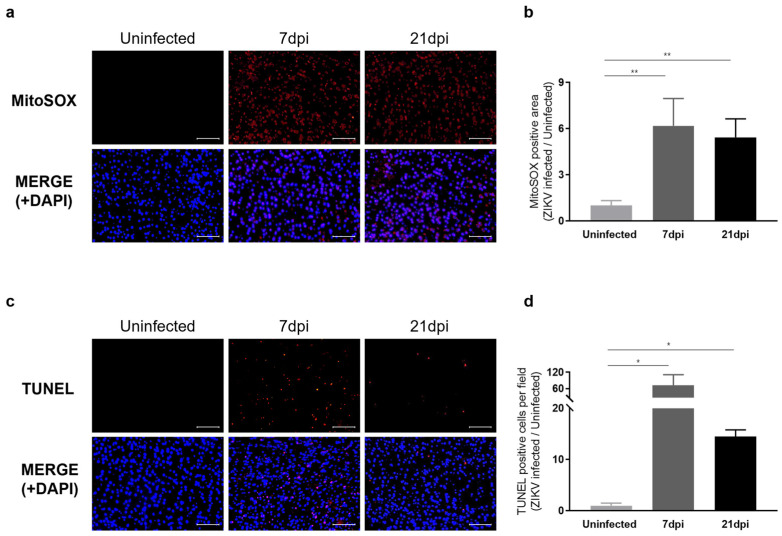
Characterization of neurological damage caused by persistent microglial activation after ZIKV Infection. (**a**) Representative images of mitochondrial ROS, stained with MitoSOX (Red), in the mouse cerebral cortex. Cell nuclei were counterstained with DAPI (blue). Images were visualized by fluorescence microscopy (200×). Scale bar: 20 μm. (**b**) Quantification of the relative MitoSOX-positive area. Results are shown as means ± SEMs of five independent replicates (** *p* < 0.01). (**c**) Representative images of DNA fragmentation (red) in the mouse cerebral cortex, detected by TUNEL assay. Cell nuclei were counterstained with DAPI (blue). Images were visualized by fluorescence microscopy (200×). Scale bar: 20 μm. (**d**) Quantification of TUNEL-positive cells in mouse cerebral cortices from five independent replicates (* *p* < 0.05).

We next examined whether sustained microglial activation following recovery from ZIKV infection results in exacerbated neuronal loss. To address this question, we evaluated histological alterations to determine the extent of pathology in mouse brain tissues. Hematoxylin and eosin (H&E) staining revealed dead neurons as eosinophilic cells with shrunken, darkly stained nuclei surrounded by a void space. In the cortex of uninfected control groups, neurons exhibited a round shape with clearly visible nuclei. In contrast, at 7 dpi, distinct neuronal damage and immune cell infiltration were observed [41], with neuronal cell death continuing through 21 dpi (Figure S5a). Additionally, Nissl staining revealed a significant decrease in both the density and number of intact neurons in the cortex at 7 dpi and 21 dpi compared to the uninfected control group (Figure S5b). These findings underscore the critical role of inflammatory responses and neuronal damage in the pathology of ZIKV infection. The inflammatory environment, including monocyte infiltration, likely contributes to the sustained activation of microglia, which, in turn, may lead to increased ROS production and further exacerbate neuronal damage. Our analysis of ROS levels in the cortex of ZIKV-infected mice supports this, revealing a significant increase in ROS compared with uninfected controls (Figure 5a). Specifically, ROS levels were approximately 6-fold higher at 7 dpi and 5-fold higher at 21 dpi compared to baseline uninfected control levels (Figure 5b). TUNEL assays conducted to assess neuronal cell death in the cortex of mice following recovery from ZIKV infection revealed a number of TUNEL-positive cells in the cortex of both ZIKV-infected and ZIKV-recovered mice (Figure 5c). A quantitative analysis showed significant differences in the number of TUNEL-labeled cells between uninfected mice and ZIKV-infected mice at both the 7 dpi and 21 dpi time points (Figure 5d). A comparison of the number of apoptotic cells in ZIKV-infected brains to uninfected controls showed a remarkable 72-fold increase in 7 dpi mice and a 14.5-fold increase in 21 dpi mice. Although the direct link between ROS generation and neuronal death should be further validated through additional studies, such as by evaluating the effects of inhibiting ROS generation, several studies have already highlighted the connection between microglial activation, ROS production, and neuronal cell death [42,43].

Consistent with transcriptome profiling results indicating that ZIKV-induced aberrant microglial activation results in neurotoxicity, histological analyses and the quantification of neuronal apoptosis revealed that ZIKV infection was associated with neurological damage in the mouse brain. Our results further indicate that even after levels of viral RNA return to near-uninfected control levels, mice recovered from ZIKV infection display neurological impairment and the activation of neuronal cell death pathways owing to the persistence of microglial activity.

### 2.6. Sustained Microglial Cell Activation Following ZIKV Infection Results in Abnormal Neural Morphology and Accumulation of Phosphorylated Tau

**Figure 6:**
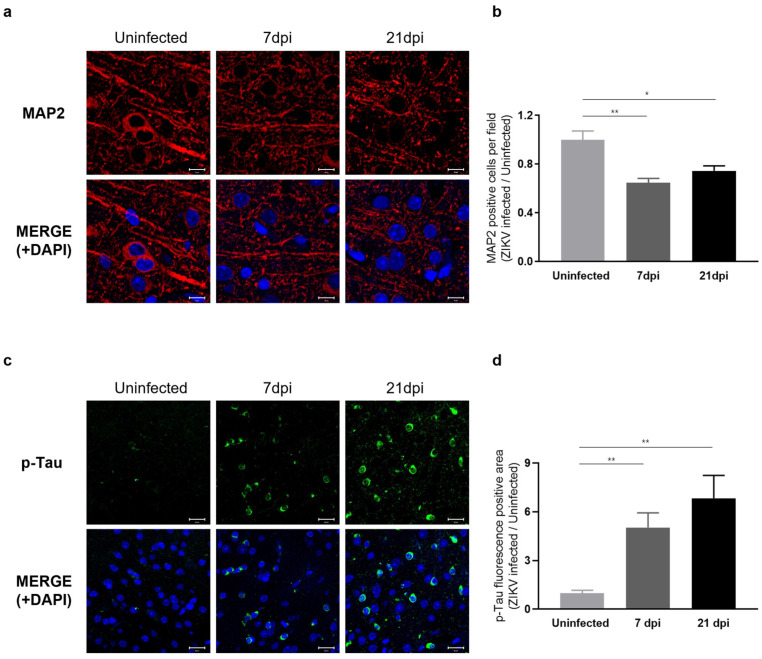
Characterization of neuronal phenotypes and Tau pathology produced by sustained microglial activation following ZIKV infection. (**a**) Representative images of MAP2 staining (red) in the mouse cerebral cortex. Cell nuclei were counterstained with DAPI (blue) and all images were visualized by fluorescence microscopy (800×). Scale bar: 10 μm. (**b**) Quantification of MAP2-positive cells. Results are shown as means ± SEMs of five independent replicates (* *p* < 0.05; ** *p* < 0.01). (**c**) Representative fluorescent images of p-Tau (PHF13, Ser396) in the mouse cerebral cortex. Cell nuclei were counterstained with DAPI (blue) and all images were visualized by fluorescence microscopy (400×). Scale bar: 20 μm. (**d**) Quantification of p-Tau fluorescence-positive area in mouse cerebral cortices from five independent replicates (** *p* < 0.01).

Our RNA-seq results indicated that ZIKV infection leads to a decrease in the expression of genes associated with axon guidance and synaptic transmission. To assess whether these transcriptomic changes manifest as neuronal phenotypes, we examined the dendritic architecture of the cerebral cortex obtained at 7 and 21 dpi by immunostaining for the neurite marker MAP2 (microtubule-associated protein 2), a neuron-specific cytoskeletal protein enriched in dendrites that plays a role in determining and stabilizing neuronal morphology during development [44]. We observed a significant reduction in the number of intact dendritic segments and branches in ZIKV-infected mice (Figure 6a), indicating a detrimental impact of ZIKV on neurite morphology. A comparison of the number of neurons in ZIKV-infected brains to uninfected baseline levels showed a 40% decrease in mice at 7 dpi and a 20% decrease at 21 dpi (Figure 6b). These findings suggest that ZIKV infection triggers the activation of microglia and the complement system, leading to excessive synaptic pruning and subsequent synaptic impairment in mice.

To gain further insights into the effects of ZIKV infection on the progression of neurodegenerative disorders, we examined Tau pathology in ZIKV-recovered mice. Under normal conditions, Tau protein stabilizes cellular microtubules, but under pathological conditions, the intracellular accumulation of hyperphosphorylated Tau disrupts microtubules and damages the cytoskeleton. This process has been linked to neurodegeneration and cognitive impairment [19,22,45]. Several studies have additionally reported that the accumulation of hyperphosphorylated Tau is associated with microglial activation [46,47,48]. Given the significance of p-Tau as a risk factor for neurodegenerative diseases, particularly Alzheimer’s disease, we conducted further evaluations of Tau protein levels using immunohistochemistry (IHC). As shown in Figure 6c, p-Tau staining was significantly increased in the cortex of ZIKV-infected mice compared with uninfected controls and further increased in ZIKV-recovered mice. These data suggest that ZIKV infection leads to sustained microglial activation and increased p-Tau levels in ZIKV-recovered mice, which may contribute to neurodegeneration (Figure 6d).

## 3. Discussion

Using a mouse model that recapitulates the recovery phase of ZIKV infection, we have elucidated the detailed mechanisms underlying the long-term sequelae of neuroinvasive ZIKV infection and provided critical evidence of a potential association with neurodegeneration. Indeed, we observed robust viral replication in the brains of these mice, peaking at 7 dpi and clearing by 21 dpi. Based on these findings, we designated 7 dpi as the acute phase and 21 dpi as the recovery phase of ZIKV infection. We then performed an integrative analysis of genome-wide gene expression changes and histological data on the ZIKV-infected mouse cerebral cortex obtained from the acute (7 dpi) and recovery (21 dpi) phases of infection. RNA-seq results revealed that sustained microglial activation plays a central role in the persistence of neuroinflammation and the downregulation of neurotransmission during the recovery phase. Persistent microglial activation is also associated with the activation of the complement system and resultant microglial phagocytosis. In line with the transcriptomic data, our histological examinations show that persistent microglial activation is associated with significant neuronal cell death and increased ROS production. Subsequent neural-cell-death-induced excessive synaptic loss and microglial-activation-mediated p-Tau accumulation imply a potential impact on neurodegeneration. 

The recent coronavirus-disease-19 (COVID-19) pandemic has highlighted the importance of investigating the long-term consequences of viral infections [49,50]. Similar to the long-term effects caused by SARS-CoV-2 and other flavivirus family members [49,51], evidence suggests that ZIKV can also cause neurological damage even after recovery from infection [8,52]. Therefore, further studies should be performed in aged mice to investigate the long-term effects of ZIKV infection and its progression towards neurodegenerative disorders. Specifically, extending the observation period to 3–6 months post infection, beyond the current 21 dpi, will be crucial for understanding the prolonged impact of ZIKV infection and its potential to induce chronic neurodegenerative changes.

Our histological data revealed that synaptic elimination was not fully restored, and p-Tau levels were even higher in the recovery phase than in the acute phase. This observation suggests that despite the apparent resolution of the acute phase of ZIKV infection, there may be ongoing pathological changes related to the accumulation of protein aggregates, such as NFTs, due to elevated p-Tau levels. Autophagy is a lysosomal degradative process that eliminates damaged organelles and protein aggregates [53]. Most neurodegenerative diseases are characterized by pathological protein aggregates that lead to the formation of NFTs due to hyperphosphorylated Tau. Impairments in autophagy are known to play a significant role in the progression of tauopathies and efficient autophagy is essential for the degradation of phosphorylated Tau, helping maintain its levels at a low threshold [54]. To further assess autophagic activity after ZIKV recovery, we performed RT-qPCR analysis on autophagy-related genes (ATGs) and autophagosome-related LC genes using ZIKV-infected brain tissues obtained at 28 dpi (Figure S6). Our results showed that autophagy-related Atg7 and Atg12 genes, as well as autophagosome-related LC3A and LC3B genes, were downregulated at 28 dpi. These genes are crucial for the elongation and closure steps of autophagosome formation, suggesting a potential impairment in the autophagic process during the recovery phase. This impairment could contribute to the accumulation of damaged proteins and cellular debris. Further studies investigating the impact of autophagy and protein aggregation on long-term recovery in mouse models of ZIKV infection could provide more insights into the relationship between autophagic defects and the pathogenesis of neurodegenerative diseases.

Several studies have demonstrated that ZIKV does not replicate efficiently in immunocompetent mice [55,56]. To more accurately model the conditions of ZIKV infection in humans, we used immunocompromised A129 mice, which are highly susceptible to ZIKV and provide valuable insights into viral replication and neuroinflammation. While A129 mice are advantageous for studying viral mechanisms, they do not fully represent how the immune system interacts with ZIKV in humans. Immunocompetent models, such as those with C57BL/6 mice, are still essential for understanding the interaction between ZIKV and a functional immune system, thereby enhancing our understanding of ZIKV pathogenesis in humans. Initially, we considered using C57BL/6 mice, a common strain of immunocompetent mice, for our experiments. These mice were intraperitoneally injected with the ZIKV-FLR strain, similar to the conditions used for A129 mice. However, we observed minimal ZIKV replication in C57BL/6 mice, which limited our ability to effectively study viral effects and pathogenesis (Figure S7). Due to these limitations, we decided to use immunocompromised A129 mice, which are highly susceptible to ZIKV. This decision allowed us to achieve more consistent and measurable viral replication, providing valuable insights into the disease. Recent research indicates that specific ZIKV strains and particular inoculation conditions can indeed induce infection in immunocompetent models [31,57]. Moreover, employing an immunocompetent mouse model with the temporal blockading of type I interferon using specific antibodies offers a promising alternative [41]. This approach allows immune responses to be elicited in immunocompetent mice while enhancing infection by temporarily blocking type I interferon. Therefore, adjusting the ZIKV strain, inoculation method, dose, and infection conditions might make it feasible to study ZIKV infection in immunocompetent mice. Additionally, there is a growing trend to use both immunocompetent and immunocompromised models to gain a comprehensive understanding of immune responses and viral pathogenesis. This approach could complement our findings and enhance the understanding of ZIKV pathogenesis in conditions that more closely resemble human infection. Therefore, incorporating both immunocompetent and immunocompromised mouse models in subsequent studies will be crucial for a comprehensive understanding of ZIKV pathogenesis, immune responses, and long-term effects. 

Moreover, although we did not observe notable behavioral abnormalities in recovered mice, incorporating specific behavioral tests for cognitive impairment and motor dysfunction might be useful in investigating neurodegenerative clinical signs in these models. Further studies to investigate the effects of diet and exercise during recovery phase would also provide valuable insights for designing new strategies to enhance recovery from ZIKV infection. 

In conclusion, we have demonstrated the sequelae of consequences of ZIKV infection in adult brain tissue using a ZIKV-susceptible recovery mouse model. Our findings provide a fundamental understanding of the in vivo mechanisms underlying brain impairments associated with ZIKV infection post recovery. Sustained microglial activation, even after recovery, may lead to persistent neuroinflammation and synaptic loss, resulting in long-term defects in neuronal function. Therefore, it is essential to develop effective antiviral vaccines to prevent ZIKV infection and to design potent therapeutics for treating ZIKV infection and related neuropathogenic flavivirus family members.

## 4. Materials and Methods

### 4.1. ZIKV Propagation 

ZIKV-MR766 strain (African lineage, accession number AY632535) and FLR strain (American and Asian lineage, accession number KU820897) were obtained from American Type Culture Collection (ATCC, Manassas, VA, USA). ZIKV was propagated in Vero cells. Briefly, Vero cells were cultured in Dulbecco’s Modified Eagle’s Medium (DMEM; Capricorn Scientific, Ebsdorfergrund, Germany) supplemented with 10% fetal bovine serum (FBS; Gibco BRL, Carlsbad, CA, USA) and 1% penicillin/streptomycin (Capricorn Scientific, Ebsdorfergrund, Germany). Cells were infected with ZIKV at a multiplicity of infection (MOI) of 0.1 for 4 days at 37 °C in a humidified chamber under 5% CO_2_ conditions. Following infection, the culture medium was harvested and centrifuged at 1000× *g* and cell supernatants were stored as virus stocks at −80 °C until needed. Infectivity was assessed by determining virus titers using a standard plaque assay in Vero cells treated with serial dilutions of virus stocks.

### 4.2. Mice

Interferon alpha/beta (IFN-α/β) receptor-deficient A129 mice were obtained from the Korea Research Institute of Chemical Technology (KRICT, Daejeon, Republic of Korea). Female A129 mice, 10–15 weeks old, were used to investigate ZIKV-induced neurological deficits. Mice in the experimental groups were intraperitoneally injected with either the ZIKV-FLR or ZIKV-MR766 strain. Specifically, we administered a challenge dose of 1 × 10^4^ PFU of the virus in 200 µL to ensure consistency and reproducibility in our infection experiments. The uninfected control group was intraperitoneally injected with an equivalent volume of phosphate-buffered saline (PBS). Mice were monitored daily for signs of illness including changes in body weight and mortality. All animal experiments were performed in a Biosafety Level 2 animal facility according to the relevant guidelines and laboratory procedures were approved by the Konkuk University Institutional Animal Care and Use Committee (IACUC).

### 4.3. RNA Isolation

Total RNA was isolated from the cerebral cortices of mouse brains using the PureLink RNA Mini Kit (Invitrogen, Carlsbad, CA, USA) according to the manufacturer’s instructions. The quantity and quality of the isolated RNA were analyzed using UV spectrophotometry (NanoPhotometer N60/N50; Implen, Munchen, Germany)

### 4.4. Quantitative Reverse Transcription-Polymerase Chain Reaction (RT-qPCR) 

cDNA was synthesized from total RNA using SuperScript III First-Strand Synthesis Supermix (Invitrogen, Carlsbad, CA, USA) and qPCR was performed on the LightCycler 96 system (Roche, Mannheim, Germany) using SsoAdvanced Universal SYBR Green Supermix (Bio-Rad, Hercules, CA, USA). Gene-specific primers used for qPCR are listed in Table S1. mRNA levels of genes were normalized to those of glyceraldehyde-3-phosphate dehydrogenase (*Gapdh*).

### 4.5. RNA Sequencing (RNA-Seq) 

Total RNA was isolated from the cerebral cortex of mouse brains and quantified with a Victor3 fluorometer (Perkin Elmer, Norwalk, CT, USA) using the PicoGreen method (Invitrogen, Carlsbad, CA, USA). The integrity of total RNA was analyzed using a 2100 Bioanalyzer (Agilent Technologies, Palo Alto, CA, USA). RNA samples with RNA Integrity Number (RIN) values ≥ 7 were used to generate RNA-seq libraries. Isolated RNA was treated with DNase to remove DNA contamination and rRNA was removed using the Ribo-Zero rRNA removal kit (Illumina, San Diego, CA, USA). Purified total RNA was randomly fragmented, synthesized into cDNA, and then used to prepare RNA-seq libraries using the TruSeq Stranded mRNA LT Sample Prep Kit (Illumina, San Diego, CA, USA) according to the manufacturer’s instructions. The quantity and quality of libraries were assessed and paired-end sequencing with an average length of 100 reads was performed using the NovaSeq 6000 system (Illumina, San Diego, CA, USA). Three replicates were used for sequencing. Complete raw and normalized RNA-seq data have been deposited in Gene Expression Omnibus (GEO) under accession number GSE265861. 

### 4.6. RNA-Seq Data Analysis

Low-quality reads were filtered out by trimming RNA-seq data using Trimmomatic (version 0.38, USA). The remaining reads were then aligned to the UCSC mouse GRCm38/mm10 reference genome using the Bowtie2 aligner (version 2.3.4.1, Johns Hopkins University, Baltimore, MD, USA). To compare expression levels across all samples, we calculated fragments per kilobase of transcript per million mapped reads (FPKM), fold change, and variance using edge R. Transcripts with FPKM > 1 were selected for further analysis. Transcripts exhibiting log2 fold change ≥ 2 and significant pairwise variance (*p* < 0.05) were categorized as differentially expressed genes. Gene set enrichment analysis (GSEA) was conducted utilizing the KEGG database and GO terms.

### 4.7. H&E and Nissl Staining

Mouse whole-brain tissues were fixed in 4% paraformaldehyde, dehydrated through a series of ethanol solutions and xylene, and embedded in paraffin according to standard protocols. The paraffin blocks were sectioned into 3–5 μm thick slices, which were then cut into coronal sections to ensure comprehensive analysis of ZIKV-induced neurological defects across all brain regions. After dewaxing and rehydration, the paraffin sections were subjected to hematoxylin and eosin (H&E) staining and Nissl staining. Neurons and Nissl bodies in the cortical region were then observed using an inverted microscope (TS2-S-SM; Nikon, Tokyo, Japan).

### 4.8. Western Blotting

Each mouse brain cortex was lysed with cold RIPA buffer (150 mM NaCl, 50 mM Tris pH 7.5, 0.1% SDS, 1% Triton X-100, 0.5% deoxycholate, protease inhibitor cocktail (Roche, Basel, Switzerland), 1 mM orthovanadate, 10 mM NaF, 100 mM PMSF) with gentle sonication. Protein samples were denatured in sodium dodecyl sulfate (SDS) sample buffer and separated by SDS-PAGE on 10% gels. The proteins were then transferred electrophoretically to a nitrocellulose membrane. Membranes were blocked with 5% non-fat milk in Tris-buffered saline with Tween 20 (TBS-T) for 1 h at 20 °C, followed by incubation with primary antibody (Table S2) overnight at 4 °C. After washing, membranes were incubated with secondary antibody (Table S2) at room temperature for 1 h. Target protein bands were visualized using enhanced chemiluminescence (Thermo Fisher Scientific Inc., Waltham, MA, USA), and signals were detected with the ChemiDoc Imaging System (Bio-Rad, Hercules, CA, USA).

### 4.9. Immunohistochemistry

For immunohistochemistry, the same paraffin-embedded brain tissues as described in Section 4.7 were used. Sections (3–5 μm) were immersed in xylene for 15 min and then sequentially rehydrated in absolute ethanol and a 95% ethanol solution in distilled water. Antigens were reactivated by treatment with 0.01 M citrate buffer for 50 min at 95 °C. Slides were washed with PBS and then incubated with primary antibodies (Table S2) overnight at 4 °C. After washing with PBS, slides were incubated with secondary antibodies (Table S2) at 25 °C for 1 h and washed twice with PBS. Slides were then covered with ProLong Gold Antifade Mountant with DNA Stain DAPI (Invitrogen, Carlsbad, CA, USA) for 1 min. Slides were imaged using an inverted microscope (Eclipse Ts2R; Nikon, Tokyo, Japan) or a confocal microscope (Zeiss LSM 800, Oberkochen, Germany) at magnifications of 400× and 600×.

### 4.10. Measurement of ROS

ROS production in brain tissue was measured by MitoSOX Red (Invitrogen, Carlsbad, CA, USA) staining according to the manufacturer’s protocol. Whole-brain tissues were rapidly frozen by immersion in dry ice and then stored at −80 °C. The frozen brain tissues were sectioned into 5–7 μm thick coronal slices. Briefly, these sections were incubated with MitoSOX Red for 15 min at 37 °C in the dark. Slides were mounted for 1 min at 25 °C in the dark using ProLong Gold Antifade Mountant with DNA Stain DAPI (Invitrogen, Carlsbad, CA, USA). ROS signals were visualized by fluorescence microscopy (Eclipse Ts2R; Nikon, Tokyo, Japan) and MitoSOX-positive areas were quantified using ImageJ software 1.54j (Media Cybernetics Inc., Rockville, MD, USA).

### 4.11. Measurement of Cell Apoptosis by TUNEL Assay

DNA fragmentation of apoptotic cells in brain sections was visualized by terminal deoxynucleotidyl transferase-mediated dUTP nick end labeling (TUNEL) staining using an in situ BrdU-Red DNA Fragmentation assay kit (Abcam, Cambridge, MA, USA) according to the manufacturer’s instructions. Slides were then counterstained with DAPI for 1 min at 25 °C in the dark. Red fluorescent signals corresponding to TUNEL-positive cells were analyzed using a fluorescence microscope (Nikon Eclipse Ts2R, Tokyo, Japan) and TUNEL-positive cells per field were quantified using ImageJ software1.54j (Media Cybernetics Inc., Rockville, MD, USA).

### 4.12. Statistical Analyses 

Results are shown as mean values ± standard errors of the mean (SEMs). Two-tailed unpaired *t*-tests were applied for statistical analysis.

## Data Availability

The data presented in this study are available from the corresponding author upon reasonable request.

## Supplementary Materials

The supporting information can be downloaded at: https://www.mdpi.com/article/10.3390/ijms25179451/s1.
