# Supplementary material for: Sustained Microglial Activation Promotes Synaptic Loss and Neuronal Dysfunction after Recovery from ZIKV Infection
Source: Int J Mol Sci. 2024 Aug 30;25(17):9451. doi: 10.3390/ijms25179451 (PMC11394746; doi:10.3390/ijms25179451)
Supplement: Supplementary file 1 [file ijms-25-09451-s001.zip › ijms-3137124-supplementary.pdf]

Supplemental Information

Sustained Microglial Activation Promotes Synaptic Loss and Neuronal Dysfunction after Recovery from ZIKV Infection

Nahyun Kim, Hanul Choi, Uijin Kim, Suyeon Kim, Young Bong Kim* and Ha Youn Shin*

*Correspondence: kimera@konkuk.ac.kr; hayounshin@konkuk.ac.kr

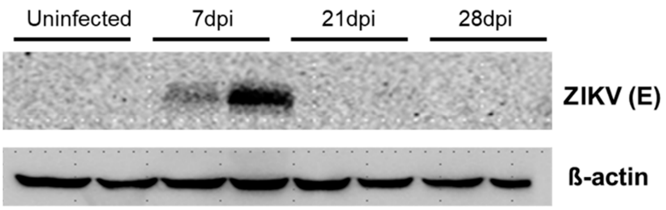

Figure S1. Expression levels of ZIKV E protein in ZIKV-infected brain tissues at different time points. Western blot analyses were performed on brain tissues obtained at 7, 21, and 28 days post-infection (dpi), with two samples per time point (n = 2 per group). The expression levels of ZIKV E protein were assessed, with β-actin used as the loading control.

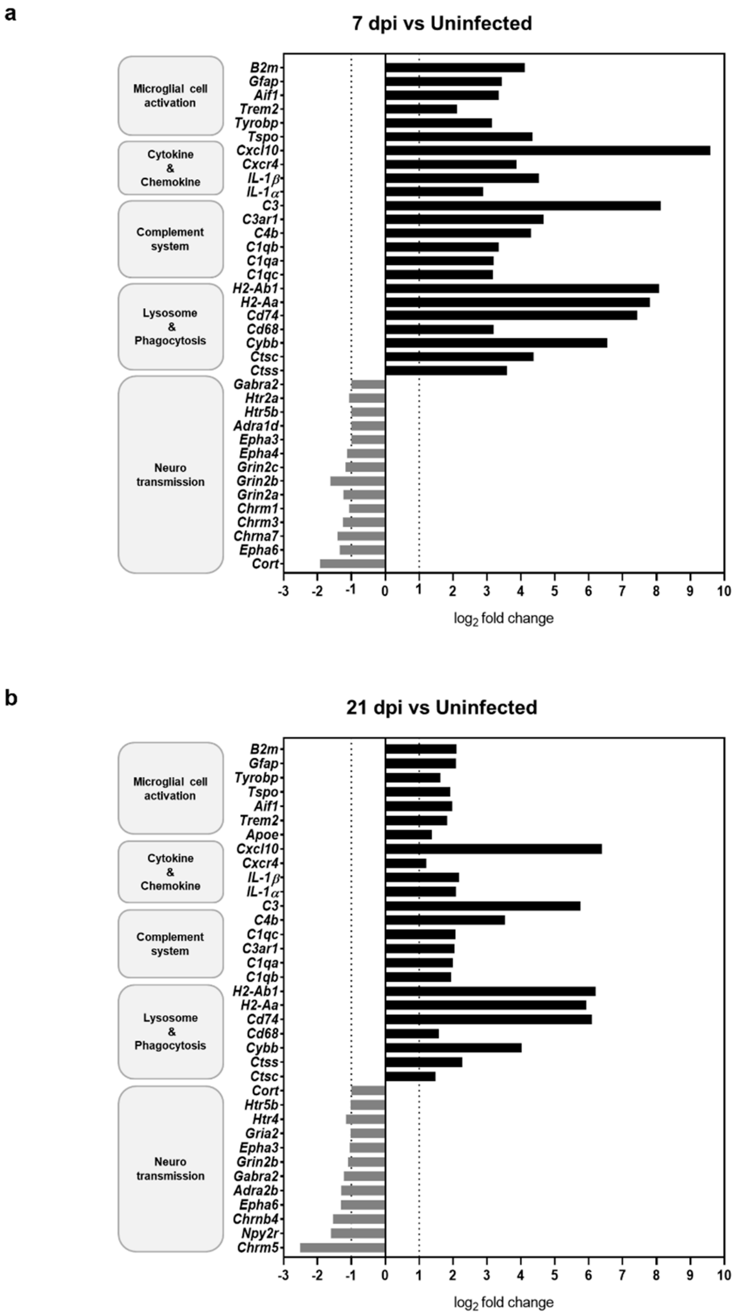

Figure S2. Transcriptional analysis of upregulated and downregulated genes in the mouse brain following ZIKV infection. (a) Log2-fold changes of significantly regulated genes ($p < 0.05$) in the cerebral cortex of ZIKV-infected mice obtained at 7 dpi. (b) Log2-fold changes of significantly regulated genes ($p < 0.05$) in the cerebral cortex of ZIKV-infected mice obtained at 21 dpi.

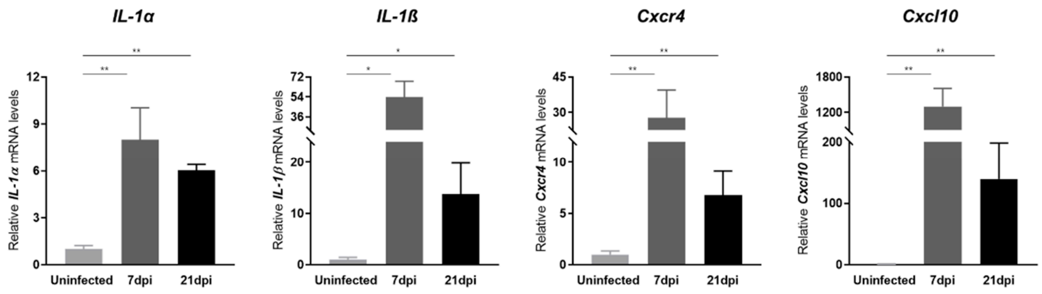

Figure S3. RT-qPCR analysis of cytokine genes. Relative mRNA levels of the cytokine genes, *IL-1α*, *IL-1ß*, *Cxcr4* and *Cxcl10*, at 7 and 21 dpi in the cerebral cortex of mice infected with ZIKV-FLR strain. mRNA levels were normalized to *Gapdh* mRNA levels. Data represent the means ± SEM of at least four independent replicates (*p < 0.05; **p < 0.01).

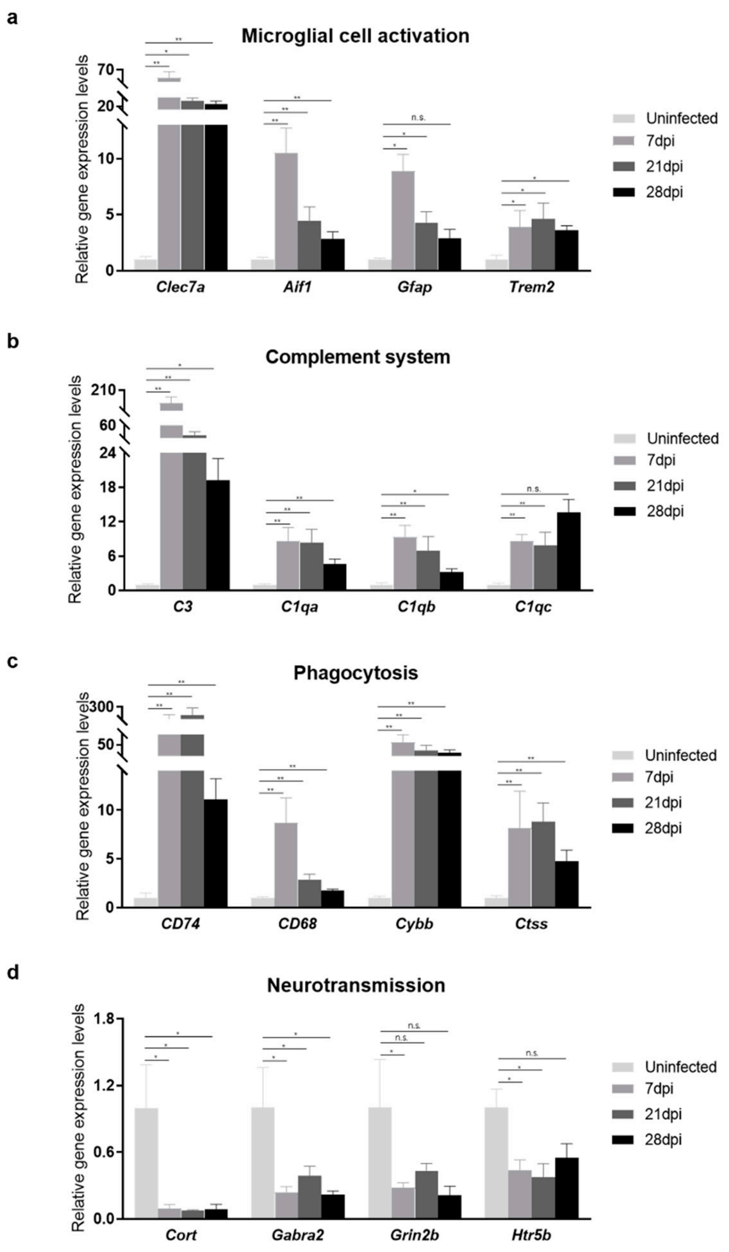

Figure S4. Transcriptional analysis of genes related to microglial activation, complement system, phagocytosis, and neurotransmission in the brains of ZIKV-infected and recovered mice. RT-qPCR analyses were performed to assess genes associated with (a) microglial activation, (b) the complement system, (c) phagocytosis, and (d) neurotransmission. mRNA levels were normalized to *Gapdh* and presented relative to uninfected controls. Data are shown as means ± SEM from at least four independent replicates (*p < 0.05; **p < 0.01; n.s., not significant).

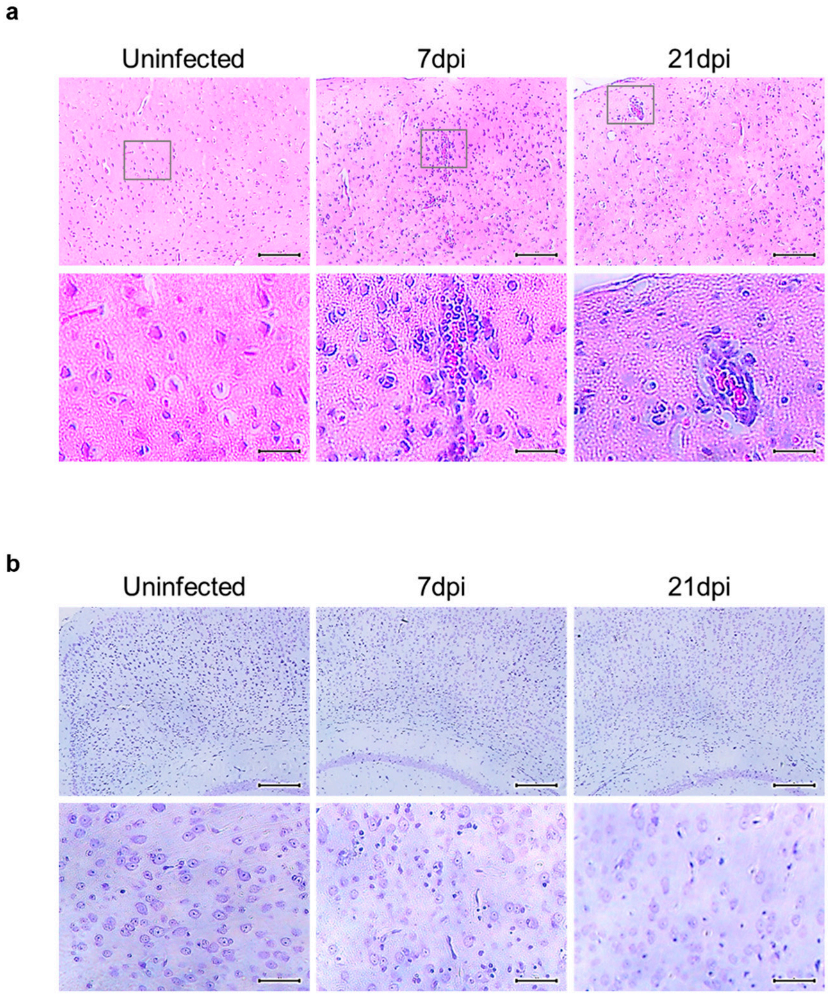

Figure S5. Histopathological analysis of ZIKV-infected mouse brain cortices. (a) Representative images of hematoxylin & eosin (H&E) staining in the cortex, with boxes highlighting areas of immune cell infiltration and nuclear shrinkage compared to uninfected controls (n = 3 per group; magnification: 40x and 400x; scale bar: 20 μm). (b) Representative images of Nissl staining in the cortex. (n = 3 per group, magnification: 40x and 200x, Scale bar, 20 μm).

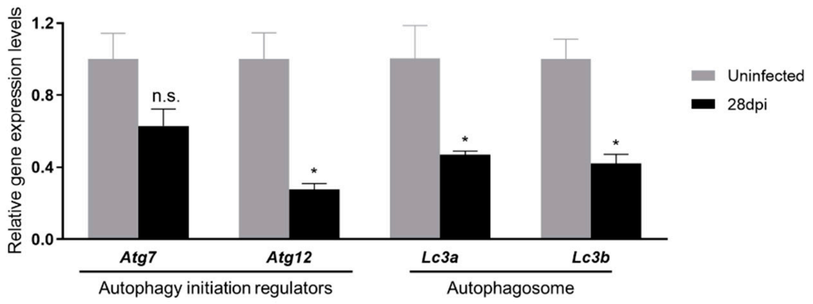

Figure S6. RT-qPCR analysis of autophagy-related genes in the cerebral cortex of ZIKV-infected mice. Relative mRNA levels of *Atg7*, *Atg12*, *Lc3a*, and *Lc3b* were measured at 28 dpi in mice infected with the ZIKV-FLR strain. mRNA levels were normalized to *Gapdh*. Data are presented as means ± SEM of four independent replicates (*$p < 0.05$; *n.s.*, not significant).

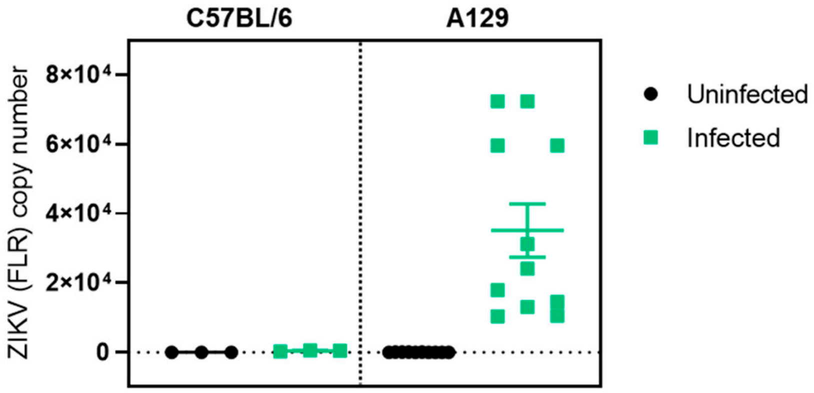

Figure S7. Comparison of ZIKV replication in the brain cortices of immunocompetent C57BL/6 and immunocompromised A129 mice. ZIKV (FLR) RNA levels in mouse brain tissues were quantified using RT-qPCR analysis. Relative ZIKV RNA levels are shown in the brains of both immunocompetent and immunocompromised mice at 7 dpi. Data are presented as means ± SEM from at least three mice per group.

Table S1. qPCR primers used in the study

Gene	Forward primer	Reverse primer
Clec7a	5’-CCAGCTAGGTGCTCATCTACTG-3’	5’-CCTTCACTCTGATTGCGGGAAAG-3’
Aif1	5’-TCTGCCGTCCAAACTTGAAGCC-3’	5’-CTCTTCAGCTCTAGGTGGGTCT-3’
Gfap	5’-CACCTACAGGAAATTGCTGGAGG-3’	5’-CCACGATGTTCCTCTTGAGGTG-3’
Trem2	5’-CTACCAGTGTCAGAGTCTCCGA-3’	5’-CCTCGAAACTCGATGACTCCTC-3’
C3	5’-AGCAGGTCATCAAGTCAGGC-3’	5’-GATGTAGCTGGTGTTGGGCT-3’
C1qa	5’-GTGGCTGAAGATGTCTGCCGAG-3’	5’-TTAAAACCTCGGATACCAGTCCG-3’
C1qb	5’-CAACCAGGCACTCCAGGGATAA-3’	5’-CCAACTTTGCCTGGAGTCCCAG-3’
C1qc	5’-AAGGACGGGCATGATGGACTCC-3’	5’-TTTCCCACGGTGGCCAGGCAT-3’
CD74	5’-GCTGGATGAAGCAGTGGCTCTT-3’	5’-GATGTGGCTGACTTCTTCCTGG-3’
CD68	5’-GGCGGTGGAATACAATGTGTCC-3’	5’-AGCAGGTCAAGGTGAACAGCTG-3’
Cybb	5’-TGGCGATCTCAGCAAAAGGTGG-3’	5’-GTACTGTCCCACCTCCATCTTG-3’
Ctss	5’-GCATAGAGGCAGACGCTTCCTA-3’	5’-CCACTGCTTCTTTCAGGGCATC-3’
Cort	5’-GGTCGCAGCCTCCGCCCTTC-3’	5’-TTGGGAAGCCCACTCGTGCCA-3’
Grin2b	5’-TTCTATCCCCGGCATCCAGCG-3’	5’-CGTGGAGCGTGGTCATTCCCA-3’
Gabra2	5’-CTCTCCCAAGTGTCATTCTGGC-3’	5’-CGAGCACTGATGCTCAAGGTTG-3’
Htr5b	5’-GTGGTGCTCTTCGTCTACTGGA-3’	5’-GGCTGTGAACACCATCTCAGAC-3’
Atg7	5’-CCTGTGAGCTTGGATCAAAGGC-3’	5’-GAGCAAGGAGACCAGAACAGTG-3’
Atg12	5’-GAAGGCTGTAGGAGACACTCCT-3’	5’-GGAAGGGGCAAAGGACTGATTC-3’
Lc3a	5’-CTGCCTGTCCTGGATAAGACA-3’	5’-CTGGTTGACCAGCAGGAAGAAG-3’
Lc3b	5’-GTCCTGGACAAGACCAAGTTCC-3’	5’-CCATTCACCAGGAGGAAGAAGG-3’
Gapdh	5’-TTGCTGTTGAAGTCGCAGGAG-3’	5’-TGTGTCCGTCGTGGATCTGA-3’

Table S2. Antibodies used in the study

Antibody	Source	Identifier
Phospho-Tau (Ser396) (PHF13) mouse mAb	Cell Signaling Technology	Cat# 9632
MAP2 rabbit pAb	Abcam	Cat# ab32454
Alexa Fluor 488 goat anti-mouse antibody	Invitrogen	Cat# R37120
Alexa Fluor 594 goat anti-rabbit antibody	Invitrogen	Cat# R37117
Anti-Zika Envelope mAb	Bio front technology	Cat# BF-1176-56
Anti-beta actin	Abcam	Cat# ab8277
Goat anti-Mouse IgG (H+L) Secondary Antibody, HRP	Invitrogen	Cat# 31430
Goat anti-Rabbit IgG (H+L) Secondary Antibody, HRP	Invitrogen	Cat# 31460
